# Dietary collagen peptides alleviate exercise-induced muscle soreness in healthy middle-aged males: a randomized double-blinded crossover clinical trial

**DOI:** 10.1080/15502783.2023.2206392

**Published:** 2023-05-03

**Authors:** Kumiko Kuwaba, Masashi Kusubata, Yuki Taga, Hiroshi Igarashi, Koichi Nakazato, Kazunori Mizuno

**Affiliations:** a Nippi Inc, Nippi Research Institute of Biomatrix, Toride, Ibaraki, Japan; b Nippi Inc, Gelatin Division, Tokyo, Japan; c Nippon Sport Science University, Research Institute for Sport Science, Tokyo, Japan

**Keywords:** Collagen peptides, muscle soreness, fatigue, muscle strength, middle-aged

## Abstract

**Background:**

Post-exercise muscle soreness and fatigue can negatively affect exercise performance. Thus, it is desirable to attenuate muscle soreness and fatigue and promote recovery even for daily exercise habits aimed at maintaining or improving health.

**Methods:**

This study investigated the effects of dietary collagen peptides (CPs) on post-exercise physical condition and fitness in healthy middle-aged adults unfamiliar with exercise. Middle-aged males (*n* = 20, 52.6 ± 5.8 years) received the active food (10 g of CPs per day) or the placebo food for 33 days in each period of the randomized crossover trial (registered at the University Hospital Medical Information Network Clinical Trials Registry with UMIN-CTR ID of UMIN000041441). On the 29th day, participants performed a maximum of five sets of 40 bodyweight squats. Muscle soreness as the primary outcome, fatigue, the maximum knee extension force during isometric muscle contraction of both legs, the range of motion (ROM), and the blood level of creatine phosphokinase (CPK) and lactate dehydrogenase (LDH) were assessed before and after the exercise load.

**Results:**

The analysis set was the per-protocol set (*n* = 18, 52.6 ± 6.0 years) for efficacy and the full analysis set (*n* = 19, 52.8 ± 5.9 years) for safety. The visual analog scale (VAS) of muscle soreness immediately after the exercise load was significantly lower in the active group than in the placebo group (32.0 ± 25.0 mm versus 45.8 ± 27.6 mm, *p* < 0.001). The VAS of fatigue immediately after the exercise load was also significantly lower in the active group than in the placebo group (47.3 ± 25.0 mm versus 59.0 ± 22.3 mm, *p* < 0.001). Two days (48 hours) afterthe exercise load, muscle strength was significantly higher in the active group than in the placebo group (85.2 ± 27.8 kg versus 80.5 ± 25.3 kg, *p* = 0.035). The level of CPK did not change over time. The level of LDH increased slightly but was not different between the groups. No safety-related issues were observed.

**Conclusions:**

These results showed that dietary CPs alleviated muscle soreness and fatigue and affected muscle strength after exercise load in healthy middle-aged males.

## 1. Introduction

Unaccustomed or intense exercise is often accompanied by muscle soreness, and the time course depends on the type, intensity, and duration of the muscle contractions, as well as on physical fitness and constitution [1]. Although the mechanism has not been elucidated in detail, muscle soreness that occurs during and immediately after exercise results from the fatigue of the skeletal muscles due to a lack of energy production, which makes it difficult for the skeletal muscle to contract [2,3]. On the other hand, muscle soreness several hours or more after exercise results from an inflammatory response following direct damage to the skeletal muscles and surrounding connective tissues by strenuous exercise involving eccentric contractions [4–6]. Muscle soreness and fatigue can negatively affect exercise performance. Therefore, it is desirable to decrease soreness and fatigue and promote recovery even for daily exercise habits aimed at maintaining or improving health.

Collagen is abundant in the skin, bones, fascia, tendons, and ligaments, and accounts for approximately 30% of the total protein in animal tissues [7]. Gelatin, collagen extracted thermally from animal tissues, is a food product with a gelling property. In recent years, collagen peptides (CPs, also referred to as collagen hydrolysate or gelatin hydrolysate), gelatin degraded with acid or enzymes to lose a gelling property, have been shown to have various beneficial effects [8–11], used as functional food ingredients, and further proposed as a sports nutrition supplement. Concerning the effects of CPs on post-exercise physical condition and fitness, Clifford et al. [12] showed that 150 drop-jumps caused muscle soreness and reduced countermovement-jump height, but after taking CPs in advance of exercise, muscle soreness probably decreased, and the recovery of jump-height accelerated. In a pilot study, Prowting et al. [13] reported that CPs intake in advance alleviated jump-height reduction after 100 drop jumps although it did not reduce the muscle soreness. These results indicate that regular intake of CPs could positively affect muscle soreness and muscle strength after intense exercise for young males regularly engaged in recreational activities or resistance training. Our interest is in the benefits of dietary CPs for middle-aged and older people who get more concerned about their health or more worried about their physical condition. Therefore, we examined the effects of dietary CPs intake on physical condition and fitness after a moderate exercise load of bodyweight squats in middle-aged males unfamiliar with exercise.

## 2. Materials and methods

### 2.1. Study design, setting, and location

**Figure 1.**
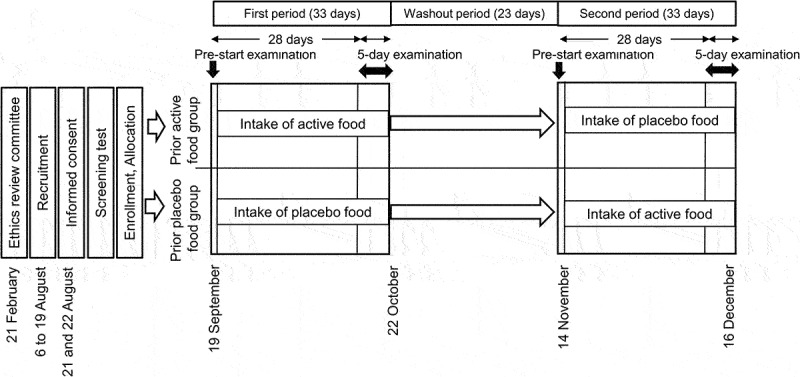
Schedule of the clinical trial.

A randomized, double-blind, crossover study was conducted by CPCC Co., Ltd. (Tokyo, Japan) of a contract research organization (CRO) under contract to Nippi Inc. (Tokyo, Japan) at the Chiyoda Paramedical Care Clinic (Tokyo, Japan) under the principal investigator physician. The study protocol was approved by the Ethics Review Committee of Chiyoda Paramedical Care Clinic (approval number: 20022102) and registered at the University Hospital Medical Information Network Clinical Trials Registry (UMIN-CTR ID: UMIN000041441). This clinical trial was carried out from 21 August to 16 December 2020 (Figure 1) and followed up until 31 January 2021. Each intervention period of this crossover trial was 33 days, including the 5-day examination at the end. The two periods were separated by the 23-day washout period.

This clinical trial was carried out from 21 August to 16 December 2020. Each intervention lasted for 33 days, with the main examinations conducted during the last 5 days. On day 1 of the 5-day examination in each period, participants performed the same set number of bodyweight squats as on the screening-test day.

### 2.2. Participants

**Figure 2.**
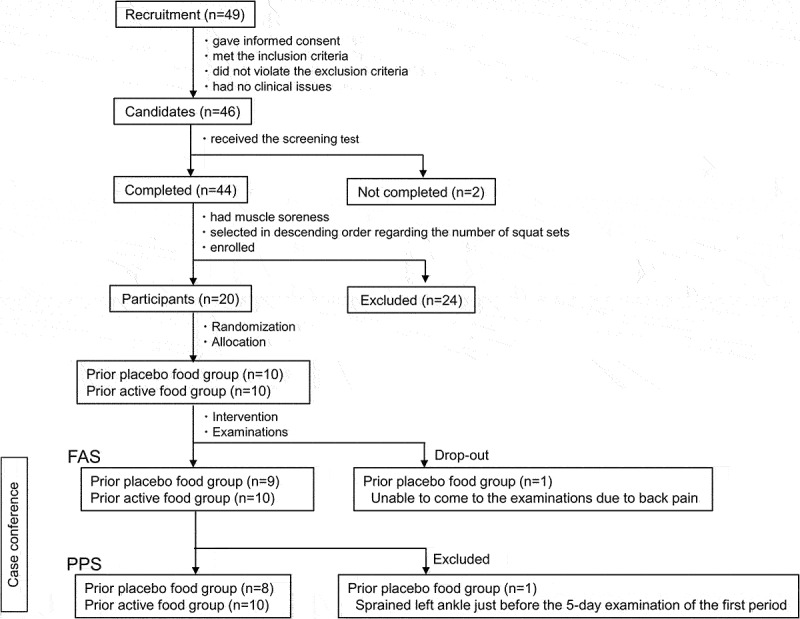
Flow chart of participants.

Participants were recruited through the Chiyoda Monitor Club (https://www.chiyo-moni.com.) (Figure 2). Forty-nine individuals visited the clinic on the screening test day. Among those who gave informed consent to participate, met the inclusion criteria, and did not violate the exclusion criteria, the principal investigator judged 46 people without clinical issues as candidates for the study. In the screening test, 44 of 46 finished bodyweight squats and recorded muscle soreness on a visual analog scale (VAS) the next day or later. Among those with muscle soreness, 20 were selected in descending order regarding the number of squat sets and enrolled. Participants lived according to the instructions. All participants were males to control for sex differences in fitness.

The inclusion criteria were as follows: (1) healthy males between 40 and 65 years of age; (2) no current regular exercise but had exercised in the past; (3) body mass index between 18.5 kg/m^2^ and 30 kg/m^2^; and (4) received a full explanation of the study, understood its contents, and gave written consent. See Appendices for the exclusion criteria and the instructions for participants.

### 2.3. Allocation and blinding

The allocation manager (LLC okutoeru, Tokyo, Japan) assigned participants randomly to two prior test food groups on a 1:1 basis using a computer-generated random number table, stratified by age, muscle strength, the number of squat sets, and the VAS of muscle soreness on the day after the screening test as allocation factors according to a central allocation method. The allocation table with subject ID and test food code was created by the allocation manager, used by the CRO to distribute the test foods to participants and then sealed until the test food representative of the sponsor unblinded the codes after the data fixation at the case conference. Thus, participants and staff of the sponsor, the CRO, the clinic, and the ethics review committee remained blinded to ensure double blindness.

### 2.4. Test foods and intervention

The active food included fish-derived CPs (Nippi Peptide Collagenomics GFF-01; Nippi Inc.) prepared using ginger protease to ensure high absorption after ingestion [14]. The placebo food included dextrin (Nippon Starch Chemical Co. Ltd., Osaka, Japan). The two test foods were calorie-matched (Table 1), white in powder, and almost colorless when dissolved in water. A total of 5 g of each test food was placed in a bag, color-coded, and provided to the CRO. The Ethics Review Committee confirmed that the two test foods were indistinguishable.

**Table 1.** Test foods.

		Active food (5 g）	Placebo food (5 g）
Nutrition	Energy	18.9 kcal	19.0 kcal
	Protein	4.6 g	0.0 g
	Carbohydrate	0.1 g	4.8 g
	Lipid	0.0 g	0.0 g
	Water	0.2 g	0.2 g
Composition	CPs	4985 mg	0 mg
	Dextrin	0 mg	4985 mg
	Flavor	10 mg	10 mg
	Sweetener	5 mg	5 mg

CPs: collagen peptides.

Participants consumed one bag of the test food twice, in the morning and evening for 33 days of each period, by dissolving one in approximately 100 mL of water. However, the timing in the morning of the 5-day examination after the 28th was followed on day 1, it was 1 hour before the exercise load (approximately 1 hour before muscle soreness assessment immediately after the exercise load), and on days 2–5, it was 1 hour before muscle soreness assessment.

### 2.5. Exercise load

On the screening test day, approximately 30 min after ingesting one salted rice ball as the prescribed meal, participants performed bodyweight squats. Instructors guided to perform bodyweight squats as follows: (1) with feet shoulder-width apart; (2) standing with hands crossed in front of the chest; (3) lowering the upper body until the knees were at 90 degrees without bending forward, followed by raising the body; (4) 40 squats of lowering the upper body in 2 seconds followed by raising the body in 2 seconds for 1 set; (5) with a 20-second rest between sets for a maximum of 5 sets. If a subject had difficulty performing the bodyweight squats or continued them in a disordered rhythm, the squats were stopped.

**Figure 3.**
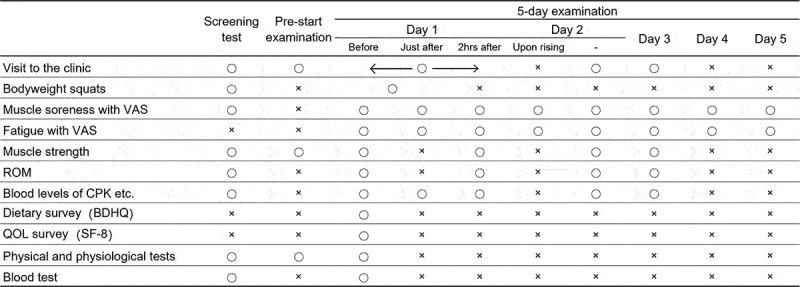
Schedule of the examinations. Before: before the exercise load, Just after: just after the exercise load, 2 hrs after: 2 hours after execise load.

On day 1 of the 5-day examination, participants performed bodyweight squats in the same set number and the same manner as on the screening test day (Figure 3).

### 2.6. Muscle soreness and fatigue

The primary outcome was muscle soreness evaluated using VAS, in which the left end (0 mm) signified no pain, while the right end (100 mm) signified the worst possible pain on a horizontal line of 100 mm. Participants recorded the perceived muscle soreness on a VAS in the 5-day examination (Figure 3). They did it after performing three squats, which were not the exercise load. Perceived fatigue was recorded on a VAS in the same manner as muscle soreness.

### 2.7. Muscle strength and range of motion

The maximum knee extension force during isometric muscle contraction of both legs was measured using a leg muscle strength measuring chair (T.K.K. 5710, Takei Scientific Instruments Co., Ltd., Niigata, Japan). Participants sat in the chair with their backs against the backrest, their knee joints at 90°, the trunk fixed with a seat belt, and their arms crossed in front of their chests. They then lifted the dynamometer attached to the chair with the knees extended. After the first measurement, a second one was after a 30-second rest. Then, a higher value was adopted.

The range of motion (ROM) of the hip joint was measured with a goniometer as participants lay on the bed, bending the knee of their right leg as close to the chest as possible.

The muscle strength and ROM were measured during the first 3 days of the 5-day examination (Figure 3).

### 2.8. The blood level of creatine phosphokinase and lactate dehydrogenase

The venous blood sample was collected on the screening test day and in the 5-day examination (Figure 3). The levels of creatine phosphokinase (CPK) and lactate dehydrogenase (LDH) were measured as markers of muscle damage.

### 2.9. Examination of intake of test food and diet

Participants recorded whether they took test food in a web-based daily logbook. On day 1 of the 5-day examination, they answered the brief-type, self-administered, diet history questionnaire (BDHQ) about their eating habits in the last month [15] (Figure 3).

### 2.10. Safety-related examinations

Participants recorded the SF-8 questionnaire on health-related quality of life in the last month. Physical & physiological tests, blood tests, and a survey on adverse events and side effects were conducted (Figure 3).

### 2.11. Statistical analysis

Efficacy and safety were evaluated using the per-protocol set (PPS) and the full analysis set (FAS), respectively (See Appendix A). Measured values for the first period in the prior active food group and the second period in the prior placebo food group belonged to the active group. The other measured values belonged to the placebo group.

A mixed model analysis of variance with group and time was used for muscle soreness VAS, fatigue VAS, muscle strength, ROM, and blood levels of CPK and LDH. Because of the non-normal distribution, the values of CPK and LDH were log-transformed. When the mixed model analysis indicated a significant interaction effect, Sidak’s multiple comparison test analyzed paired values between the test food groups at each time point, followed by the determination of the 95% confidence interval (CI) of the mean difference and effect size. When the mixed model analysis indicated a time effect, Dunnett’s test analyzed the repeated values. Because of the crossover trial, significant inter-group differences were valid in the case of neither carryover effects nor period effects (See Appendix A).

The baseline characteristics of participants were compared between the prior-test food groups using t-tests. For intake rate of test food, BDHQ, and SF-8 surveys, as well as physical & physiological and blood tests, paired t-tests compared values between the test food groups. As for the intra-group comparison, paired t-tests were used for blood tests and the SF-8 survey, and repeated Dunnett’s test for physical & physiological tests. The incidence of adverse events and side effects were analyzed using Fisher’s exact test. All data are shown as means ± SD.

The significance level was 5% with a two-tailed test. Microsoft Excel (Microsoft Corp., Redmond, WA, USA) was used for tabulation and graphing, and IBM® SPSS 26.0 (IBM Japan, Tokyo, Japan), Microsoft Excel, and GraphPad Prism 6 (GraphPad Software, San Diego, CA, USA) were used for statistical analyses.

In this trial, the effect size for muscle soreness was assumed to be 0.8 based on the previous study [16], and the sample size with a significance level of 0.05 and power of 0.8 was estimated to be 14 for the paired comparison. The number of participants was 20 to support dropouts and exclusions.

## 3. Results

### 3.1. Participants

One subject of the prior placebo food group was a drop-out because he did not visit the clinic for the 5-day examination of the first period due to back pain, and another of the prior placebo food group was excluded from the analysis set because he had sprained the left ankle just before the 5-day examination of the first period, resulting in the FAS of 19 participants and the PPS of 18 participants (Figure 2). Regarding the baseline characteristics at the screening test, there were no significant differences between the prior-test food groups in age, physical & physiological tests, muscle strength, number of squat sets, and muscle soreness VAS on the following day (Table 2).

**Table 2.** Baseline characteristics of participants.

		Prior placebo food			Prior active food			p-value
Age	years	53.6	±	7.3	51.9	±	5.0	0.562
Height	cm	166.1	±	4.6	169.8	±	5.2	0.141
Weight	kg	65.7	±	7.8	65.5	±	3.5	0.963
BMI	kg/m^2^	23.8	±	2.4	22.8	±	1.8	0.356
Soft lean mass	kg	47.5	±	4.8	46.6	±	2.4	0.604
Body fat	%	23.2	±	4.0	24.5	±	3.5	0.488
BP systolic	mmHg	122.5	±	13.4	124.2	±	14.1	0.799
BP diastolic	mmHg	77.8	±	7.2	81.9	±	9.9	0.338
Heart rate	bpm	68.1	±	7.5	71.6	±	9.3	0.405
Muscle strength	㎏	72.3	±	36.4	68.1	±	18.0	0.755
Number of squat set	sets	5.0	±	0.0	5.0	±	0.0	-
Muscle soreness VAS	mm	59.9	±	23.2	58.7	±	27.6	0.921

The maximum knee extension force during isometric muscle contraction of both legs was measured using a leg muscle strength measuring chair. Participants performed a maximum of five sets of 40 bodyweight squats. Muscle soreness was assessed with VAS on the following day of bodyweight squats.

p-value is for inter-group comparisons by *t*-test.

Prior placebo food group: *n* = 8; Prior active food group: *n* = 10.

Data are presented as mean ± SD. BMI: body mass index, BP: blood pressure.

### 3.2. Muscle soreness and fatigue

**Figure 4.**
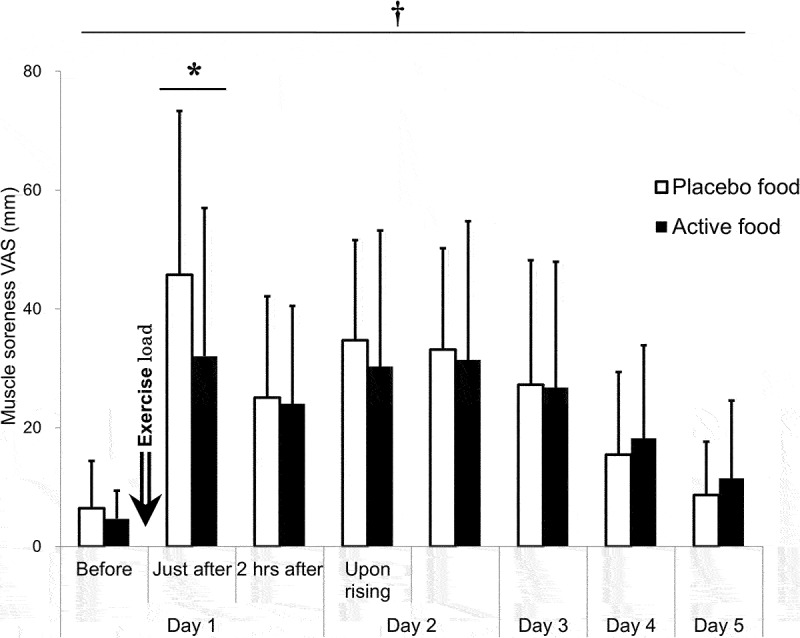
Muscle soreness VAS.

A time effect (*p* = 0.002) and an interaction effect (*p* = 0.032) were on the VAS of muscle soreness. The VAS of muscle soreness peaked immediately after the exercise load and was significantly higher until day 4 than pre-exercise. Inter-group comparisons at each time point showed that the VAS was significantly lower in the active group (32.0 ± 25.0 mm) than in the placebo group (45.8 ± 27.6 mm) immediately after the load (*p* < 0.001, mean difference: 13.7 mm, 95% CI: 4.2~23.2 mm, Cohen’s d = 0.678) (Figure 4 and Table 3). Neither carryover nor a period effect was between the prior test food groups at that time (Table S1).

Perceived muscle soreness was evaluated with VAS. The active group: n = 18; the placebo group: n = 18. †: p < 0.05 for an interaction effect by the mixed model analysis. *: p < 0.05 by Sidak’s multiple comparison test. Before: before the exercise load, Just after: just after the exercise load, 2 hrs after: 2 hours after the exercise load.

**Table 3.** Muscle soreness VAS.

			Day 1									Day 2						Day 3			Day 4			Day 5		
			Before			Just after			2 hrs after			Upon rising			-											
Mean ± SD (mm)		Active	4.7	±	4.7	32.0	±	25.0	24.0	±	16.5	30.3	±	22.9	31.4	±	23.4	26.8	±	21.2	18.2	±	15.7	11.5	±	13.1
		Placebo	6.5	±	8.0	45.8	±	27.6	25.1	±	17.0	34.7	±	16.8	33.2	±	17.0	27.3	±	20.9	15.5	±	13.9	8.7	±	8.9
p-value	Mixed Model	Group effect	0.056																							
		Time effect	0.002																							
		Interaction effect	0.032																							
	Dunnett’s test		-			<0.001			<0.001			<0.001			<0.001			<0.001			0.012			0.676		
	Sidak’s multiple comparisons test		>0.999			<0.001			>0.999			0.830			>0.999			>0.999			0.988			0.987		
Difference		Mean				13.7																				
		95% CI				4.2	∼	23.2																		
Cohen’s d						0.678																				

the active group: *n* = 18, the placebo group: *n* = 18.

Before: before the exercise load, Just after: just after the exercise load, 2 hrs after: 2 hours after the exercise load.

Difference: values of the placebo group minus the active group.

A time effect (*p* = 0.004) and an interaction effect (*p* = 0.012) were found on the VAS of fatigue. The VAS was significantly higher from the point just after the exercise to day 3, compared with pre-exercise. Inter-group comparisons at each point showed that the VAS was significantly lower in the active group (47.3 ± 25.1 mm) than in the placebo group (59.0 ± 22.3 mm) immediately after the load (*p* < 0.001, mean difference: 11.7 mm, 95% CI: 4.0~19.4 mm, Cohen’s d = 0.715) (Table 4). At this point, neither a carryover effect nor a period effect was found between the prior test food groups (Table S2). Two hours after the exercise load, the VAS was still significantly lower in the active group than in the placebo group, but a carryover effect was present (Table 4 and Table S2).

**Table 4.** Fatigue VAS.

			Day 1									Day 2						Day 3			Day 4			Day 5		
			Before			Just after			2 hrs after			Upon rising			-											
Mean ± SD (mm)		Active	4.8	±	4.1	47.3	±	25.0	28.6	±	20.2	26.4	±	21.7	23.3	±	19.6	18.8	±	17.2	14.2	±	14.1	9.4	±	11.0
		Placebo	5.6	±	6.0	59.0	±	22.3	37.0	±	20.7	33.6	±	15.9	25.5	±	13.3	19.6	±	15.4	12.1	±	9.9	6.3	±	6.9
p-value	Mixed Model	Group effect	0.338																							
		Time effect	0.004																							
		Interaction effect	0.012																							
	Dunnett’s test		-			<0.001			<0.001			<0.001			<0.001			<0.001			0.053			0.917		
	Sidak’s multiple comparisons test		>0.999			<0.001			0.025			0.084			0.991			>0.999			0.992			0.906		
Difference		Mean				11.7			8.4																	
		95% CI				4.0	∼	19.4	0.7	∼	16.1															
Cohen’s d						0.715			0.510																	

the active group: *n* = 18, the placebo group: *n* = 18.

Before: before the exercise load, Just after: just after the exercise load, 2 hrs after: 2 hours after the exercise load.

Difference: values of the placebo food group minus the active food group.

### 3.3. Muscle strength and ROM

**Figure 5.**
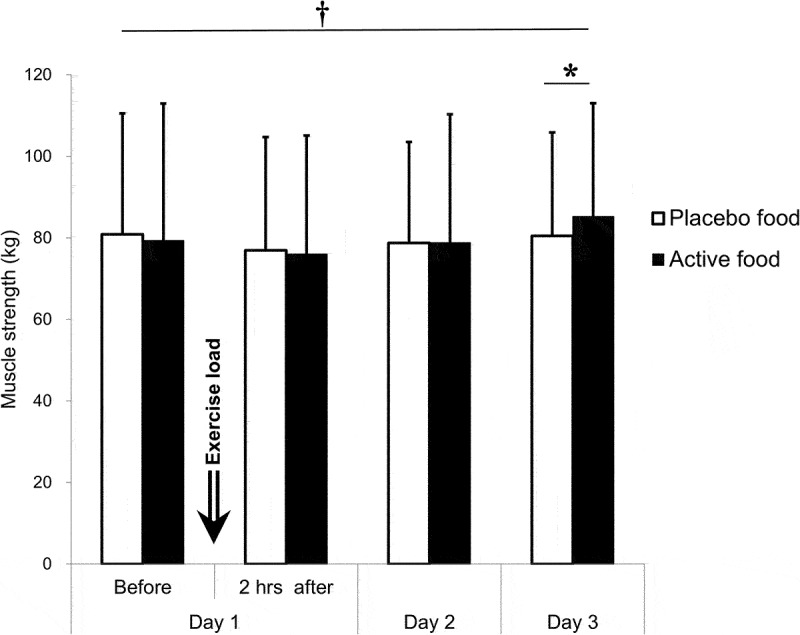
Muscle strength.

A time effect (*p* = 0.005) and an interaction effect (*p* = 0.034) were on muscle strength. Despite the time effect, the muscle strength had no variation over time. Inter-group comparisons at each point showed that the muscle strength was significantly higher in the active group (85.2 ± 27.8 kg) than in the placebo group (80.5 ± 25.3 kg) on day 3, 48 hours after the exercise load (*p* = 0.035, mean difference: −4.8 kg, 95% CI: −9.3~-0.2 kg, Cohen’s d = 0.460) (Figure 5 and Table 5). At this point, neither a carryover nor a period effect was found between the prior test food groups (Table S3).

The maximum knee extension force was evaluated using a leg muscle strength–measuring chair. The active group: n = 18; the placebo group: n = 18.

†: p < 0.05 for an interaction effect by the mixed model analysis.

*: p < 0.05 by Sidak’s multiple comparison test. Before: before the exercise load, Just after: just after the exercise load, 2 hrs after: 2 hours after the exercise load.

**Table 5.** Muscle strength.

			Day 1						Day 2			Day 3		
			Before			2 hrs after								
Mean ± SD (kg)		Active	79.3	±	33.6	76.0	±	29.1	78.8	±	31.5	85.2	±	27.8
		Placebo	80.8	±	29.7	76.9	±	27.7	78.8	±	24.7	80.5	±	25.3
p-value	Mixed Model	Group effect	0.636											
		Time effect	0.005											
		Interaction effect	0.034											
	Dunnett’s test		-			0.076			0.778			0.211		
	Sidak’s multiple comparisons test		0.866			0.972			>0.999			0.035		
Difference		Mean										−4.8		
		95% CI										−9.3	∼	−0.2
Cohen’s d												0.460		

Before: before the exercise load, 2 hrs after: 2 hours after the exercise load.

Difference: values of the placebo food group minus the active food group.

the active group: *n* = 18, the placebo group: *n* = 18.

Neither a group effect, a time effect, nor an interaction effect was on ROM (Table 6).

**Table 6.** Range of motion.

			Day 1						Day 2			Day 3		
			Before			2 hrs after								
Mean ± SD (∘)		Active	99.1	±	7.8	100.9	±	7.4	99.9	±	7.7	98.5	±	7.1
		Placebo	98.8	±	7.9	101.0	±	8.2	99.0	±	7.6	98.7	±	8.7
p-value	Mixed Model	Group effect	0.715											
		Time effect	0.781											
		Interaction effect	0.620											

the active group: *n* = 18, the placebo group: *n* = 18.

Before: before the exercise load, 2 hrs after: 2 hours after the exercise load.

### 3.4. The blood levels of CPK and LDH

Neither a group effect, a time effect, nor an interaction effect was on the CPK level. The time effect (*p* < 0.001) was at the level of LDH. It increased slightly and significantly immediately after the exercise load to day 3 (48 hours) after exercise load (Table 7).

**Table 7.** Blood levels of CPK and LDH.

			Day 1									Day 2			Day 3		
			Before			Just after			2 hrs after								
CPK																	
Mean ± SD (U/L)		Active	133	±	47	142	±	47	134	±	44	168	±	61	160	±	52
		Placebo	134	±	63	143	±	67	134	±	60	169	±	67	168	±	64
p-value	Mixed Model	Group effect	0.865														
		Time effect	0.648														
		Interaction effect	0.709														
LDH																	
Mean ± SD (U/L)		Active	173	±	29	192	±	25	179	±	27	180	±	27	181	±	28
		Placebo	171	±	24	190	±	26	177	±	22	182	±	24	183	±	27
p-value	Mixed Model	Group effect	0.633														
		Time effect	<0.001														
		Interaction effect	0.335														
	Dunnett’s test		-			<0.001			0.002			<0.001			<0.001		

the active group: *n* = 18, the placebo group: *n* = 18.

Before: before the exercise load, Just after: just after the exercise load, 2 hrs after: 2 hours after the exercise load.

### 3.5. Test food intake rate and dietary survey

The intake rate of the test food did not differ between the test food groups and was close to 100% (data not shown). The BDHQ survey showed no inter-group differences in the intakes of energy, total protein, animal protein, or vegetable protein during the intervention period (Table S4).

### 3.6. Safety-related examinations

The SF-8 survey showed no deterioration in the health-related quality of life (Table S5). Several inter-group differences and variations over time were observed in physical & physiological tests and blood tests, but these were all within the reference value ranges (data not shown). Although several adverse events were noted, there was no significant difference in the incidence rates. All adverse events were neither severe nor related to the intake of test food, and no side effects were noted (Table S6).

## 4. Discussion

This study was conducted to examine the effects of dietary CPs intake on physical condition and fitness after unaccustomed exercise in healthy middle-aged individuals. The results showed that muscle soreness and fatigue immediately after the exercise load were alleviated by CPs intake. Moreover, dietary CPs intake affected muscle strength 48 hours post-exercise.

Unaccustomed or intense exercise is often accompanied by muscle soreness [1]. Although the mechanisms underlying the development of muscle soreness are not well understood, muscle soreness during or immediately after exercise is due to fatigue of the skeletal muscles caused by insufficient energy production, which results in difficulty in contracting skeletal muscles [2,3]. In contrast, muscle soreness that occurs several hours or more after exercise is due to an inflammatory response related to direct damage to the skeletal muscles and surrounding connective tissues by strenuous exercise involving eccentric contractions [4–6]. In the present study, muscle soreness occurred over a similar time course to perceived fatigue; it was stronger immediately than the day after the exercise load. CPK levels were much lower than those reported in studies examining other exercise loads [12,17]. Therefore, direct damage and subsequent muscle soreness were slight in this trial. Rather, the bodyweight squats of one set of 40 repetitions of 4-second bodyweight squats for a maximum of 5 sets with an interval of 20 seconds caused muscle soreness and fatigue immediately, and continuous pre-exercise CPs intake relieved them. In general, exercise performance depends on several factors, but it is ultimately limited by fatigue and subsequent muscle soreness [18]. The present data suggested that dietary CPs intake might enable individuals to perform the prescribed exercise with ease and improve exercise performance. In addition to muscle soreness and fatigue, dietary CPs intake positively affected the lower limb muscle strength 48 hours post-exercise (on day 3).

The two clinical trials examined the effects of CPs intake on endpoints after strenuous exercise in recreationally active or resistance-trained young males. One article [12] showed that 150 drop-jumps caused muscle soreness and reduced countermovement-jump height, and that consumption of CPs for 1 week appeared to decrease muscle soreness after 48 hours and accelerated recovery of jump height after 48 hours. The other [13] reported a pilot study showing that consuming CPs for 1 week alleviated the countermovement jump-height reduction after 100 drop-jumps but not muscle soreness. Therefore, these and our trials showed certain benefits for post-exercise physical condition and fitness. However, the following points should be notable. The performance of 100 or 150 drop-jumps in the previous studies reduced muscle strength, but the bodyweight squats in the present study did not. More importantly, in the present study, muscle soreness occurred more strongly immediately after the exercise load than the following day. These results in the present study must be related to what we purposely set the bodyweight squats as a comparatively moderate exercise load for middle-aged people unfamiliar with exercise.

It is uncertain how dietary CPs intake decreased post-exercise fatigue and subsequent muscle soreness in this clinical trial, but it is worth discussing as below. In the animal model for depression research, we found that intake of CPs significantly prolongs forced swimming time in mice, which is caused by dopamine receptors in part [19]. The prolongation indicates an antidepressant-like effect of the test substance but also suggests a state of swimming with ease. Dietary CPs may then have acted on brain tissue to relieve muscle soreness following the perceived fatigue after exercise. For further consideration, the bodyweight squats in this trial must have reduced a certain amount of glycogen, but not a large amount, because the energy substrate for ATP production is fat and glycogen at a moderate exercise intensity [18]. Our previous studies suggested that in mice reared on CPs-containing feed, fatty acid oxidation in liver tissue is enhanced [20], and fatty acid synthesis in the liver and white adipose tissue is decreased, with a reduction in adipocyte size [21]. Therefore, we would consider that continuous CPs intake enhanced lipolysis in the liver or adipose tissue, supplying more free fatty acids to the skeletal muscle, saving glycogen, and keeping the rate of ATP synthesis to alleviate fatigue and muscle soreness immediately after exercise. Although related data are limited and the discussion is highly speculative, dietary CPs might act on brain tissue and fat metabolism to affect the post-exercise physical condition.

As for the mechanism underlying increment in muscle strength several factors are considered to contribute: the mass and the properties of skeletal muscle; the activation status of motor nerves; the availability of ATP; and the function of surrounding connective tissues such as the fascia and tendons [18,22,23]. As already discussed, continuous CPs intake might have promoted lipolysis in the liver or adipose tissue, finally resulting in maintaining ATP supply. Related to the surrounding connective tissue, our previous animal studies suggested that consumption of CPs-containing feed improves the mechanical properties of the Achilles tendon and fascia [24,25]. If these changes occur in the surrounding connective tissue, they might affect muscle strength. However, further studies are needed to support these speculations.

CPs are hydrolyzates of gelatin, which is heat-extracted animal collagen. CPs consist of a repeating (glycine (Gly)-X-Y) n amino acid sequence, where X and Y are frequently proline (Pro) and hydroxyproline (Hyp), respectively. Gly accounts for one-third of the composition, and the imino acids, Pro and Hyp, account for one-fifth [26]. The N-terminal peptide bonds of imino acids are less susceptible to hydrolysis; thus, ingested CPs are absorbed in the blood as not only free amino acids but also Hyp-containing oligopeptides. The Hyp-containing peptide detected at the highest concentration in the blood after CPs ingestion is Pro-Hyp [27,28]. In the present study, free Hyp and peptidic Hyp including Pro-Hyp were detected in blood samples after CPs intake (data not shown). The *in vitro* biological activities of Pro-Hyp are shown by several studies [29,30]. As for our in vitro studies of Pro-Hyp relevant to the considerations above, 3T3-L1 mouse adipose progenitor cells with supplementing Pro-Hyp result in smaller fat droplets showing larger total surface areas [31], representing a state of increased efficiency of lipolysis [32]: Pro-Hyp promotes the differentiation of mouse tendon progenitor cells in addition to tendon cell proliferation and collagen fiber network formation [33]. Therefore, in conjunction with the previous discussion, we would hypothesize that Pro-Hyp is at least one of the components responsible for the beneficial effects of CPs intake on post-exercise physical condition and fitness, although this hypothesis is speculative and requires further research to substantiate.

## 5. Limitations

The present trial has potential limitations. First, muscle soreness with VAS as the primary outcome was significantly lower in the active group than in the placebo group immediately after the exercise load. At this point, the power was 0.60 and the sample size for 0.8 of power was 28. Second, despite targeting middle-aged individuals, only males were selected as study participants due to sex-related differences in fitness.

## 6. Conclusion

For middle-aged individuals, dietary CP intake could alleviate exercise-induced muscle soreness and fatigue and could improve the muscle strength, possibly enabling exercise with greater ease to improve exercise performance. The dietary CPs appear to be an efficient and safe sports nutritional supplement. Further studies, including the underlying mechanism and sex-related differences with a large sample size, are needed.

## Supplementary Material

Supplemental Material

## Data Availability

The datasets are available from the corresponding author on reasonable request.

## Abbreviations

BDHQ: brief-type, self-administered, diet history questionnaire
CI: confidence interval
CPK: creatine phosphokinase
CPs: collagen peptides
CRO: contract research organization
FAS: full analysis set
Gly: glycine
Hyp: hydroxyproline
LDH: lactate dehydrogenase
PPS: per-protocol set
Pro: proline
ROM: range of motion
VAS: visual analog scale.

## Appendices Appendix A.

### Exclusion criteria

The exclusion criteria were as follows: (1) previous or current medical history of serious diseases of the circulatory, respiratory, gastrointestinal, urinary, or endocrine systems; (2) routinely using pharmaceutical products or quasi-pharmaceutical products that may affect the study results, or unable to stop such use during this clinical trial; (3) allergy to medicine or food (including collagen or gelatin); (4) routinely consuming dietary supplements that may affect the study results or unable to stop such consumption during this clinical trial; (5) preferring collagen-rich foods such as fish skin or beef tendon and consuming them at least 4 times a week; (6) engaged in any exercise or physical activity followed by muscle soreness for 1 month prior to the screening-test; (7) planned to engage in any exercise or physical activity that could cause muscle soreness during this trial; (8) regularly receiving treatments such as massage, osteopathy, chiropractic, or acupuncture, or unable to refrain from such treatments during the period of this clinical trial; (9) unable to participate according to the schedule of this clinical trial; (10) smoker; (11) heavy alcohol drinker; (12) extremely irregular eating habits or an irregular life schedule, such as with shift workers or late-night workers; (13) participating in other clinical trials have finished doing so within 4 weeks, or planned to do so after providing informed consent for this study.; (14) donated 200 mL of component blood or whole blood in the month prior to the start of the study; (15) donated 400 mL of whole blood within 3 months prior to the start of the study; (16) total volume of blood collected in the 12 months prior to the start of this study plus that planned to be collected in the present study, exceeding 1200 mL; (17) judged ineligible to participate in the study by the principal investigator physician.

### Instructions for participants

Participants were required to stick to the following: (1) record information in a web-based daily logbook (on intake of test food, meal, collagen-rich meals, healthy supplementary food, and alcoholic drink. On changes in exercise, sleep, lifestyle, and physical condition. On medical visits, treatment, and use of medicines); (2) record information in a dietary survey chart for 4 days just before the start of the intake in each period; (3) not make changes in lifestyle habits; (4) not perform exercises that could affect the study results; (5) not go on a diet and not overeat; (6) refrain from treatments, such as massage, osteopathy, chiropractic, or acupuncture; (7) refrain from taking dietary supplements; and (8) refrain from eating a collagen-rich diet.

### FAS and PPS criteria

The FAS was the population of all study participants except those who did not consume the test foods and for whom no post-assignment data existed. The Per Protocol Set (PPS) was the population of the FAS except for those participants as follows: those whose intake rate was below 85% during at least one of the first and second periods; those who repeatedly failed to follow the instructions for participants; those whose results were deemed unreliable based on conspicuous behaviors such as noncompliance with fasting or smoking cessation; those who were found after the trial to have met the exclusion criteria; and those who gave clear reasons for dropping out of the study.

### Carryover and period effects

For the carryover effect, the sum obtained by adding measured values of the first period to those of the second period was analyzed using *t*-tests between the prior-test food groups. For the period effect, the difference obtained by subtracting measured values of the second period from those of the first period in the prior-active food group and the difference obtained by subtracting measured values of the first period from those of the second period in the prior-placebo food group were analyzed using *t*-tests between the prior-test food groups [34].
